# PeVL1 Novel Elicitor Protein, from *Verticillium lecanii* 2, Enhances Systemic Resistance against Rice Leaf Roller (*Marasmia ruralis* Wlk.) in Rice (*Oryza sativa* L.)

**DOI:** 10.3390/microorganisms11020317

**Published:** 2023-01-26

**Authors:** Khadija Javed, Yong Wang, Humayun Javed

**Affiliations:** 1 Department of Plant Pathology, Agriculture College, Guizhou University, Guiyang 550025, China; 2 Faculty of Mountain Agriculture and Environmental Sciences, Kohsar University Murree, Murree 47150, Pakistan; 3 Julius Kühn-Institut (JKI) for Biological Control, 243 64287 Darmstadt, Germany; 4 Rothamsted Research West Common Harpenden, Hertfordshire AL5 2JQ, UK

**Keywords:** *Verticillium lecanii* 2, *Marasmia ruralis*, *Oryza sativa*, larval developmental instars, fecundity, expressions of defense-related genes

## Abstract

The hazardous pest known as rice leaf roller (*Marasmia ruralis* Wlk.) (Lepidoptera: Pyralidae), which undermines rice (*Oryza sativa* L.) output globally, folds the leaves of the rice plant. Protein elicitors are thought to be biological elements that causes the rice to become resistant to herbivores. The potential for biocontrol of the emerging elicitor protein evaluated from *Verticillium lecanii* 2 (PeVL1) was evaluated against *M. ruralis*. To assess the impact of PeVL1 on immature development, survival, and lifetime, four different PeVL1 concentrations were allocated. Electrical penetration graphs (EPGs) against *M. ruralis* were used to evaluate adult reproductive efficiency and the interaction between the pest and the pathogen. Furthermore, the characterization of active substances in PeVL1 with multi-acting entomopathogenic effects looked into the direct interactions of PeVL1 with temperature and climatic change in rice (*O. sativa*) plants. PeVL1 treatments reduced the population increase of second and third generation *M. ruralis* compared to controls. In a test of host selection, *M. ruralis* colonized control plants more quickly than PeVL1-treated *O. sativa* plants. PeVL1 concentrations prolonged the *M. ruralis* larval stage. Similar to fecundity, PeVL1-treated seedlings produced fewer offspring than control seedlings. On PeVL1-treated leaves, trichomes and wax production created an unfavorable habitat for *M. ruralis*. PeVL1 changed the surface structure of the leaves, which inhibited colonization and decreased *M. ruralis* reproduction. The activation of pathways was another aspect of systemic defense activities including jasmonic acid (JA), salicylic acid (SA), and ethylene (ET). Based on these results against *M. ruralis*, the use of PeVL1 in the agroecosystem with integrated pest management and biocontrol seems appropriate. Our research provides a novel insight into a cutting-edge biocontrol method utilizing *V. lecanii* 2.

## 1. Introduction

Successful pathogens must be able to recognize and overcome host plant defense responses. Plants have evolved resistance (R) proteins in response to pathogens that avoid, tolerate, or suppress basal defense, resulting in gene-for-gene resistance. Plants’ ability to resist disease is due to a combination of inducible and constitutive defense mechanisms [1]. To name microbes or pathogens, researchers use conserved, essential compounds. Innate immunity recognizes them via pattern recognition receptors (PRR) [2]. These raise extracellular pH, cause oxidative bursts, make nitric oxide (NO), secondary metabolites, and hypersensitive responses (HR) [1,2]. These resistance responses begin in cells near the infection site. As a result, the plant develops systemic acquired resistance (SAR) against a wide range of pathogens [3]. Plants need two built-in defense mechanisms. For example, flagellin, a pathogen associated molecular pattern (PAMP), helps plants identify microbes and pathogens. PAMP activates the plant’s innate defense system via a number of plant trans-membrane PRRs on the plant’s surface [4]. Gene-for-gene resistance is a type of defense that occurs mostly inside plants. In this case, the pathogen-secreted elicitors are compared to R proteins [5]. As part of effector-triggered immunity, R-proteins trigger hypersensitive responses, oxidative stress, NO production, extracellular pH elevation, cell wall augmentation, and pathogen-related protein expression (ETI) [3,5]. This type of response starts at the infection site and spreads to nearby uninfected cells. This allows the plant to fight pathogens more effectively [6]. 

Elicitors, in both biotic and abiotic ways, are responsible for stimulating the defense response and mechanism of action in plants [7]. Elicitors help bacteria, viruses, oomycetes, and fungi to grow [8]. Induced proteins, lipids, and carbohydrates are some of the compounds which help plants fight disease [9]. A common link between HR and reactive oxygen species (ROS), such as H_2_O_2_ and O^2^, is ion influx. These molecules act as elicitor signaling molecules [10]. Elicitors are classified as race-specific or universal defense groups that affect both host and non-host plants [11]. In order to ensure food safety, some chemical pesticides may be replaced by elicitors [12,13,14,15,16,17,18]. Aphid defense responses have been examined in many aphid–plant interactions. Aphid resistance in *Brassica napus* (Brassicaceae) reduced the endurance proportion and population progression of immature *Plutella xylostella* L. (Lepidoptera: Plutellidae) [19]. Plants that inhale jasmonic acid, salicylic acid, and ethylene strengthen their defenses. Aphids respond to JA and SA [20,21,22], and aphids eating JA–SA increase the expression of defense-related genes [23].

Numerous microorganisms, such as entomopathogenic fungi (EPF), have demonstrated their efficacy against a variety of insect pests [24]. In addition to this, EPF possess the ability to produce endophytes within a variety of plant tissues [25]. EPF promote plant development and create systemic resistance to biotic stressors in several plants, including pathogens and phytoparasites [26]. They also boost yield [27], enhance the nutritional value of plants [28], and encourage the growth of plant roots [29]. In broth cultures, many EPF have been described to produce a wide variety of insecticides, anti-feedants, and bio-actively poisonous compounds [30]. Local and systemic defense responses have been observed in host plants when recombinant PeVL1 from the entomopathogenic fungus *V. lecanii* 2 was applied. The JA and SA pathways increase plants’ resistance to it. Our study is focused on the purification and characterization of a novel protein elicitor, PeVL1, that was extracted from an entomopathogenic fungus called *V. lecanii* 2 strain and its impact on *M. ruralis* management. Additionally, we are interested in the potential bioactivity of this protein elicitor against a pest called *M. ruralis*, which feeds on *O. sativa* L. The findings of this study will contribute to and reveal information about the development of a potentially novel strategy for the biological control of *M. ruralis*.

## 2. Materials and Methods

### 2.1. Establishment of M. ruralis Colonies and O. sativa Cultivation

The focus of this research was to grow *M*. *ruralis* and *O. sativa* colonies in a controlled growing season before the tests. *Marasmia ruralis* was found nearby *Triticum aestivum* L., and was transferred to seedlings of *O. sativa* (Gailiangmaofen802F1, Jiaxin Seed Limited Company). During the experiment, a colony of *M*. *ruralis* was kept at room temperature for 6 months before the experiment. *Marasmia ruralis* were reared on *O. sativa* plants at 26 ± 2 °C and a relative humidity of 70–80% with a photoperiod time of 10 D:14 L. The seeds of *O. sativa* were cleaned in 75% ethanol for 30 s, before being washed and soaked in water for 2–3 days prior to using.

### 2.2. PeVL1 Evaluation

PeVL1 was expressed in *Escherichia coli* BL21-DE3 using the recombinant vector pET30- *Bam*HI and *Hind*III (Novagen, Darmstadt, Germany), purified using a HisTrap™ HP column (GE Healthcare, Boston, MA, USA), and desalted in a HiTrap desalting column ™ (GE Healthcare, Waukesha, WI, USA) as described by Nam et al., 2020 [31].

### 2.3. Marasmia ruralis Infestation on the Plant

This experiment was designed to estimate the size of the *M. ruralis* population that had settled. *Oryza sativa* were soaked in 89.24 μg mL^−1^ PeVL1 for one day. Four organic seeds were developed (Flora Guard substrate). After seven days, *O. sativa* seedlings were sprayed with 89.24 μg mL^−1^ PeVL1 solution and inoculated with 15 *M. ruralis* adults. Seedlings were treated weekly. Every five days after inoculation, *M. ruralis* were counted. The data fractions were used to analyze and comprehend the data. Controls and negative controls were 89.24 μg mL^−1^ water and 50 mM Tris-HCl at pH 8.0. Plants were caged in transparent, breathable mesh. Each time, four replicates were used.

### 2.4. Growth Rate of M. ruralis

The purpose of this experiment was to see if feeding PeVL1-treated or control seedlings enhanced the intrinsic growth of *M. ruralis*. *Oryza sativa* seeds were managed as in (Section 2.3). Seeds were splattered with 89.24 μg mL^−1^ of PeVL1 pure protein solution after a day. A glass tube lined with cotton gauze isolated the sprouts, and *M. ruralis* mobility was restricted on the leaf using a plastic ecological cage (2.7 × 2.7 × 2.7 cm). To prevent mechanical damage to the leaf, the perimeter of the ecological cage was sponge-coated. Every 12 h, the *M. ruralis* larval instar was checked for larvae production. In order to avoid crowding, newly molted larvae were counted two times every day to govern the total aggregate of time and offspring created. This was done again five days later on seeds and plants. Thirty duplicates of each treatment were used in the experiment. The intrinsic rate was calculated as follows:

*r_m_* = 0.738 × (ln *Md*)/*Td*

*Md* counts the set of newborn larvae in a *Td* development phase (the period of time among an *M. ruralis* infancy at first reproduction).

### 2.5. Marasmia ruralis Bioassay

This study’s goal was to examine *Marasmia ruralis* larval development and fertility. On *O. sativa* plants, PeVL1 was tested against *Marasmia ruralis* at 89.24, 53.54, 26.77, and 13.38 μg mL^−1^. This was calculated using the Bradford assay. Using a separate spray bottle, approximately 3 mL of PeVL1 was sprayed onto the *M. ruralis* plants at the three-leaf stage. Water and buffer were used to treat the controls (50 mM Tris-HCl, pH 8.0). Between 3-6 *Marasmia ruralis* with ages of 0–6 h old were given to *O. sativa* plants which were desiccated instantaneously. The total amount of descendants formed by all *M. ruralis* larval instars was used to calculate the overall larval development period, while *M. ruralis* longevity was derived from the number of days lived. The bioassays were performed in triplicate at three different temperatures (23, 25, and 27 °C).

### 2.6. PeVL1 Impacts on O. sativa Development and Structure

The goal of this study was to see how PeVL1 affected *O. sativa* growth and structure. *Oryza sativa* seeds and seedlings were treated as described in Section 2.3. A 3.5 percent glutaraldehyde solution in 0.1 M phosphate solution was used to collect samples for up to two days (pH 7.2). In total, five 2 h submersions in 1 percent osmic acid were performed on all samples. It was used for 15 min with an ethanol gradient of 100% to 95% to 90% to 80% to 70% to 30%. EM critical point drier Leica was used to dry all critical points (CPD030; Leica Bio-systems, Wetzlar, Germany). Samples were then inspected with a Hitachi H-7650 TEM. PeVL1-treated colonies were measured in 10 duplicates.

### 2.7. HPLC/MS

The goal of this study was to quantify the amount of SA, JA, and ET accumulated in this way; seeds and seedlings were handled as described previously. Seedlings’ aerobatic sections were collected for SA, JA, and ET sampling [32]. This was done using an HPLC/MS (Shimazu Research Instruments, ODS-C18, 3 m, 2.1 per 150 mm, Kyoto, Japan). Methanol mobile phase, 60 percent, and 4 °C sample temperature were used in HPLC. Sim system in negative ion mode was used with the following parameters: solvent 250 °C, heat block 200 °C, gas flow rates 10 L/min, nebulizing gas 1.5 L/min, detector voltage 1.30 kV, and interface 3 kV (SA *m*/*z*: 137.00; JA: 209.05).

### 2.8. Gene Expressions

TransGen Biotech (Beijing, China) kits extract RNA, synthesize cDNA, and perform qRT-PCR (ABI 7500 Real-Time PCR System). The RNA was tested using an NP80 nano-photometer. *LOC_Os12g37350.1*, *LOC_Os11g39220.1*, *LOC_Os06g23760.1*, *LOC_Os08g39850.1*, *LOC_Os11g15040.4*, *LOC_Os01g56380.1*, *LOC_Os03g53200.1*, *LOC_Os05g41210.1*, *LOC_Os11g08380.1*, *LOC_Os03g01130.1*, *LOC_Os01g10940.1*, *LOC_Os03g37710.1* were tested for the JA, SA and ET pathway. The internal reference was the 18S ribosomal gene [33]. Table 1 lists the primers used. The relative fold expression of genes was assessed using the 2^−ΔΔCT^ method [34].

### 2.9. Data Analysis

Using Statistix software version 8.1, ANOVA and Leven’s tests were used to compare two treatments, while LSD and ANOVA compared three or more treatments (Tallahassee, FL, USA). These data were first square-root transformed before analysis. To eliminate disparities, we used a 95 percent probability LSD test on treatment variables such as PeVL1 elicitor concentrations and temperature regimes. For gene expressions, the comparative CT (2^−∆∆CT^) method was used; the fold changes having protein elicitor and buffer applied were compared at (α = 0.05)

## 3. Results

### 3.1. Marasmia ruralis Indoors

**Figure 1:**
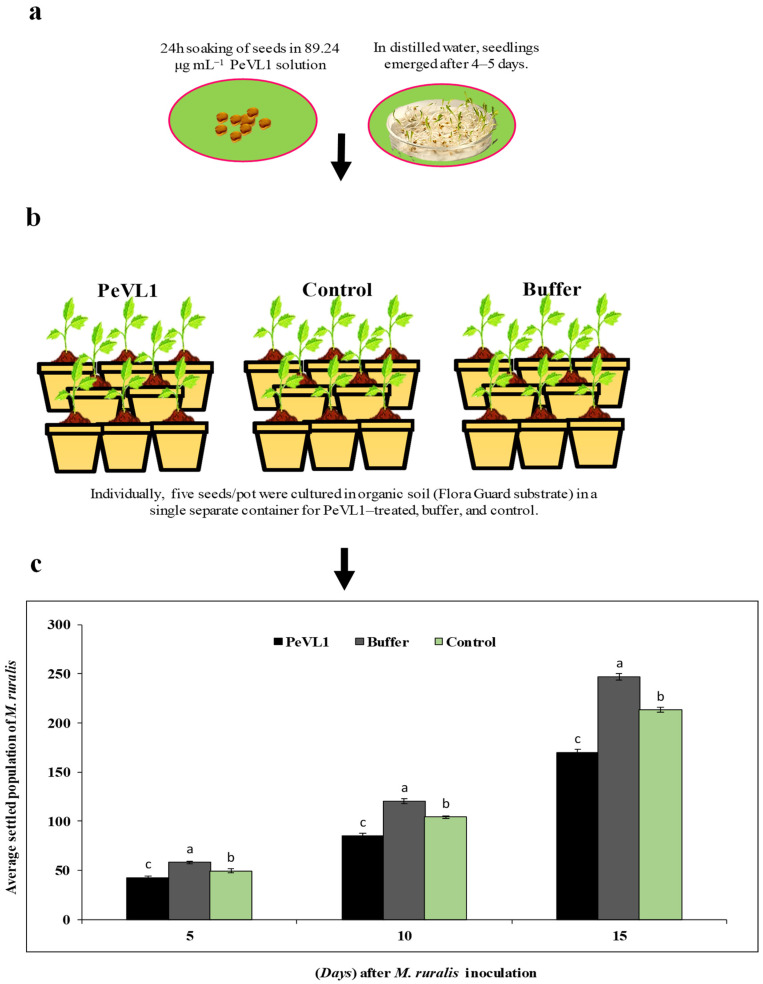
*Marasmia ruralis* population differences were observed in PeVL1-, control-, and buffer-treated *O. sativa* seedlings. Using one-way ANOVA, Levene’s test with SPSS 18.0, the LSD at *p* = 0.05. (**a**,**b**) Seeds and seedling treatment. (**c**) Seedlings treated with PeVL1 saw a substantial *M. ruralis* population loss (mean ± SD). (**a**–**c**) small letters on each bar are significant differences among treatments and control.

**Figure 2:**
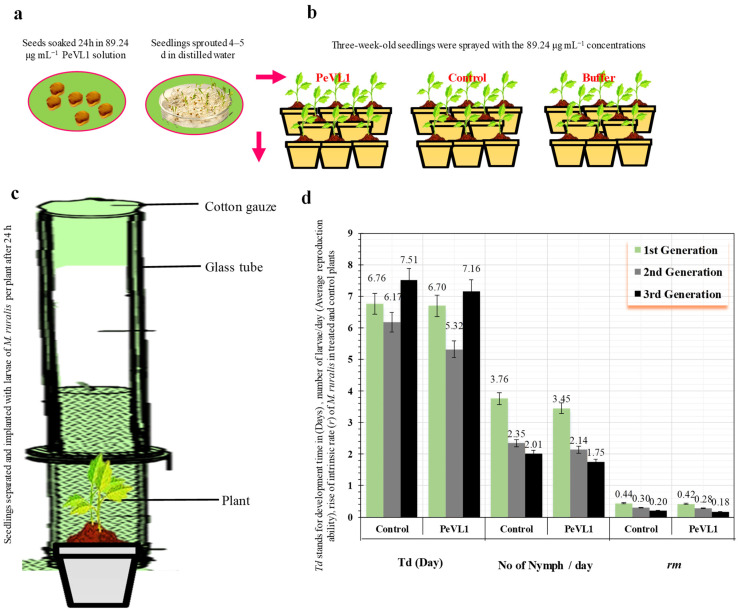
*Marasmia ruralis* development time in *O. sativa* seedlings treated with PeVL1 and control, (**a**,**b**) Seeds and seedling treatment (Mean ± SD), (**c**) seedlings treated with PeVL1 and control in glass tube (**d**) Seedlings treated with PeVL1 saw a substantial change of generation in *M. ruralis* population (mean ± SD) the study used SPSS 18.0 using LSD and one-way ANOVA at *p* = 0.05.

PeVL1 induced *M. ruralis* resistance in two ways. *Marasmia ruralis*-treated *O. sativa* seedlings had significantly reduced *M. ruralis* populations. Figure 1 compares the population declines in the PeVL1 treatment to the buffer and control. When *M. ruralis* fed on PeVL1-treated seedlings, their everyday reproductive potential was reduced; all generations showed lower growth rates, according to the findings (Figure 2).

### 3.2. PeVL1 Influenced M. ruralis Larval Development and Fecundity

**Figure 3:**
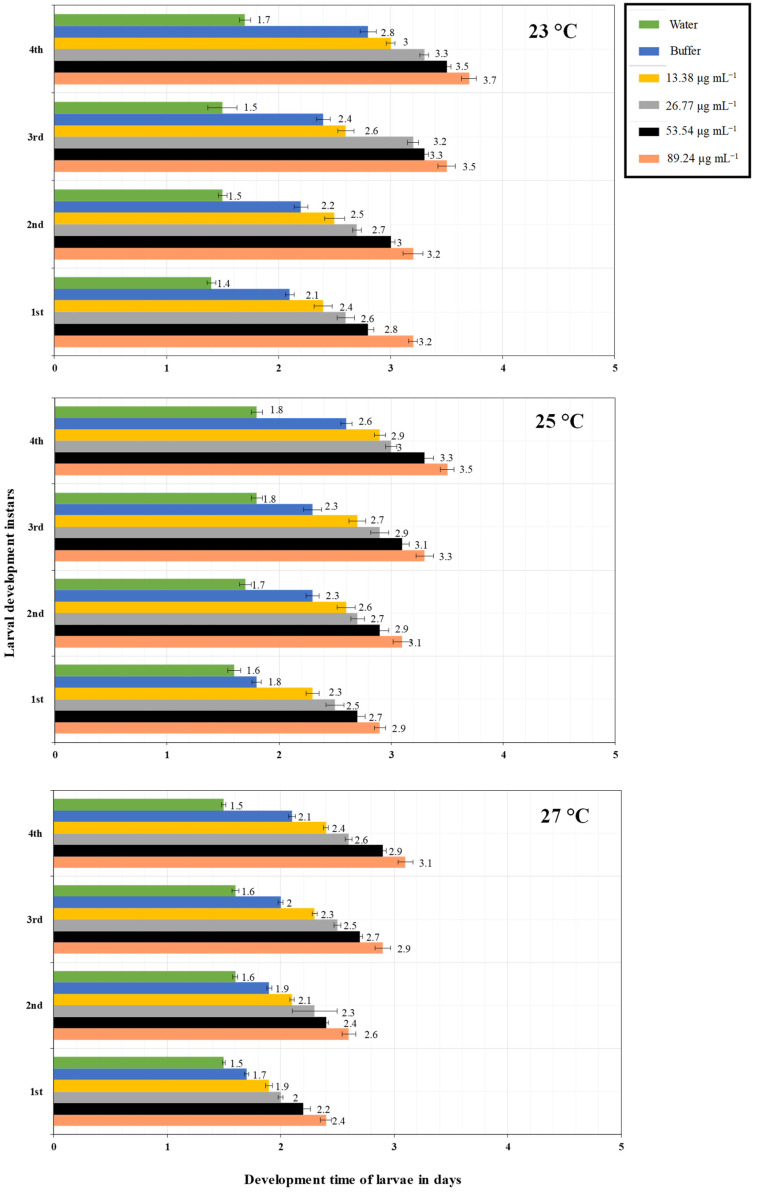
On *O. sativa* plants, the PeVL1 elicitor protein elicited the development of *M. ruralis* larval instars at various doses and temperatures (23, 25, 27 °C) (*n* = 10; one-way ANOVA with factorial analysis; LSD at α = 0.05).

**Figure 4:**
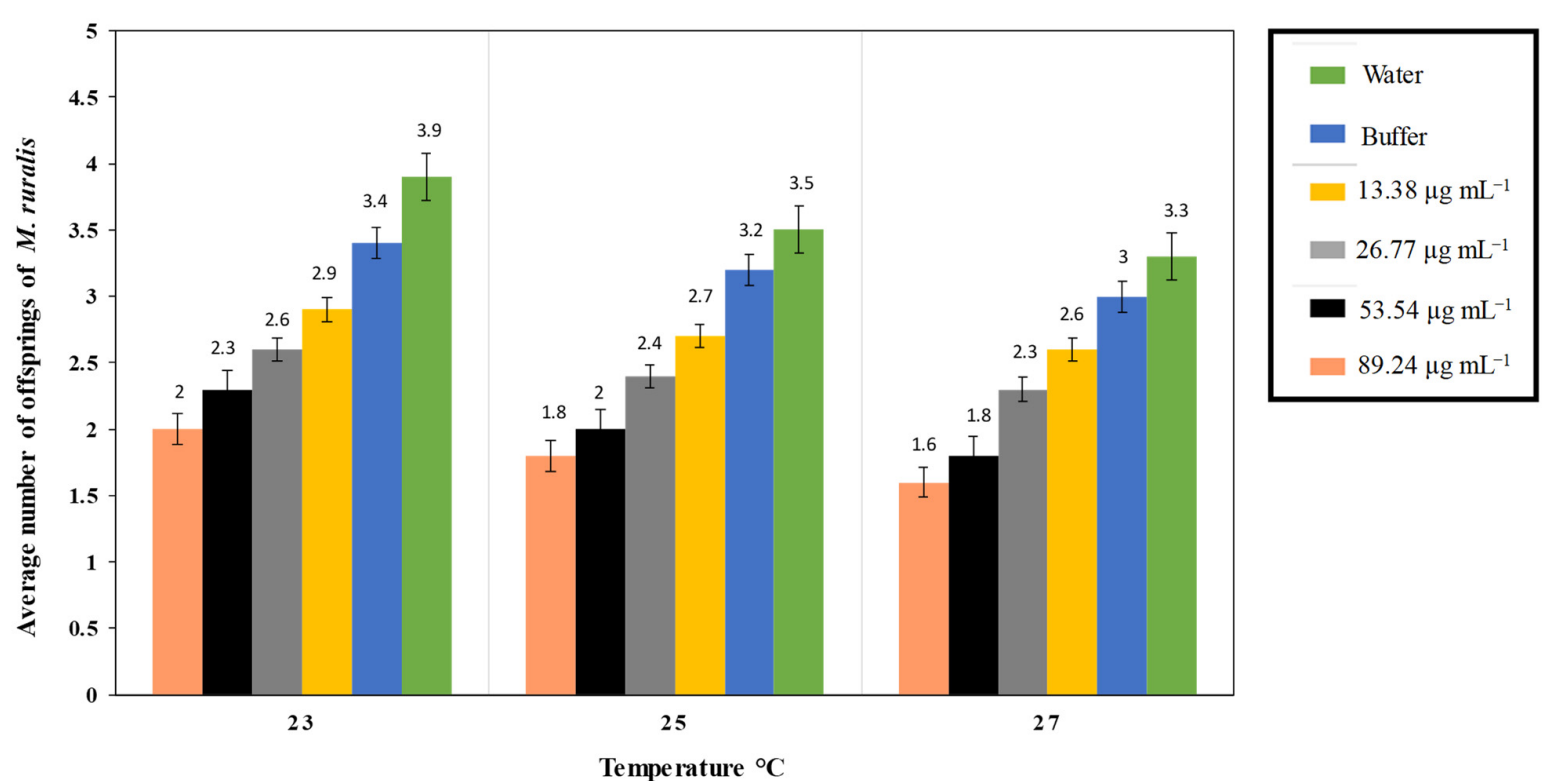
*Marasmia ruralis* average fecundity (*n* = 10), fecundity lessened in *O. sativa* seedlings treated with PeVL1, (one-way ANOVA with factorial analysis; LSD at α = 0.05).

The interaction of different PeVL1 concentrations with three temperature regimes influenced the overall development period of *M. ruralis*. As PeVL1 concentrations increased, so did larval instar development time (Figure 3). Fourth larval instar development time was 3.7 days at 89.24 µg ml^−1^ and 23 °C; a concentration of 13.38 µg ml^−1^ at 27 °C produced a minimum of 1.5 days larval growth. *Marasmia ruralis* fecundity influenced PeVL1 concentrations and temperatures (Figure 4). The experiment found that fecundity was lowest at 27 °C and highest at 23 °C.

### 3.3. PeVL1 Influenced the Development and Structure of O. sativa

**Figure 5:**
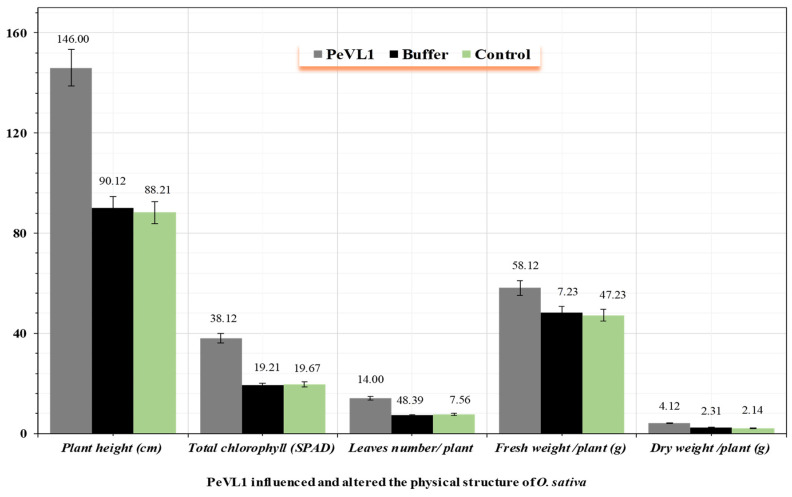
The effect of PeVL1 on the growth of treated and untreated seedlings. (*n* = 10) PeVL1 and buffer seedling (mean ±SD). The data were compared with LSD and one-way ANOVA with Levene’s test (*p* = 0.05) in SPSS 18.0.

PeVL1 significantly influenced the plant height and surface structure of *O. sativa* seedlings compared to control seedlings (Figure 5). PeVL1-treated seedlings had significantly more trichomes than control seedlings, with (96.10 ± 0.58 mm^−2^ in PeVL1-treated seedlings versus 34.15 ± 0.31 mm^−2^ in control seedlings; *p* = 0.05). With a better surface environment and a more complex wax structure, *M. ruralis* colonization should be more difficult.

### 3.4. SA, JA, and ET Quantities

**Figure 6:**
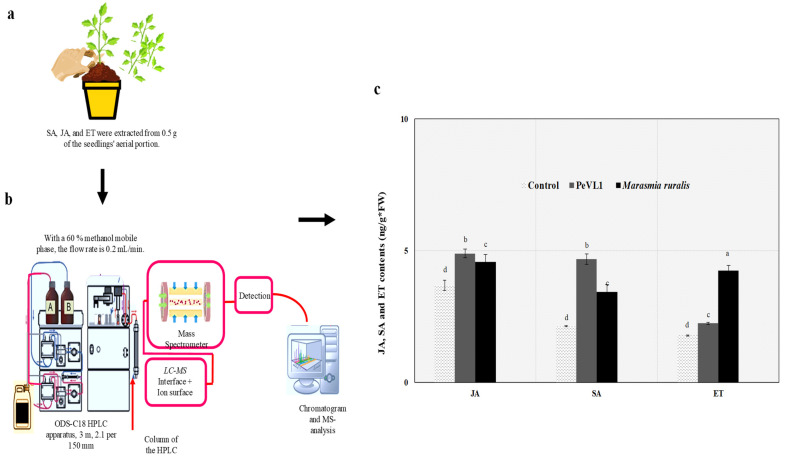
*Oryza Sativa* seedling JA, SA, and ET levels (mean ± SD). (**a**,**b**) An additional day after spraying, PeVL1 data were collected. (**c**) *Marisma ruralis* were infected in both treatments. The LSD, ANOVA, and Leven’s test were used to compare data. Lower-case letters indicate significant JA, SA, or ET treatment differences. (*p* = 0.05).

We examined links between JA, SA, and ET and cuticular wax deposition, trichome number, and *M. ruralis* infestation on PeVL1. Content of JA, SA, and ET was found to be higher in PeVL1-treated seedlings (Figure 6). The development of *M. ruralis* resistance in *O. sativa* requires all three signaling pathways. The protein elicitor elicited an innate immunological or defensive response in *O. sativa* plants, according to the findings.

### 3.5. Defense-Related Gene Expression Fold Change

**Figure 7:**
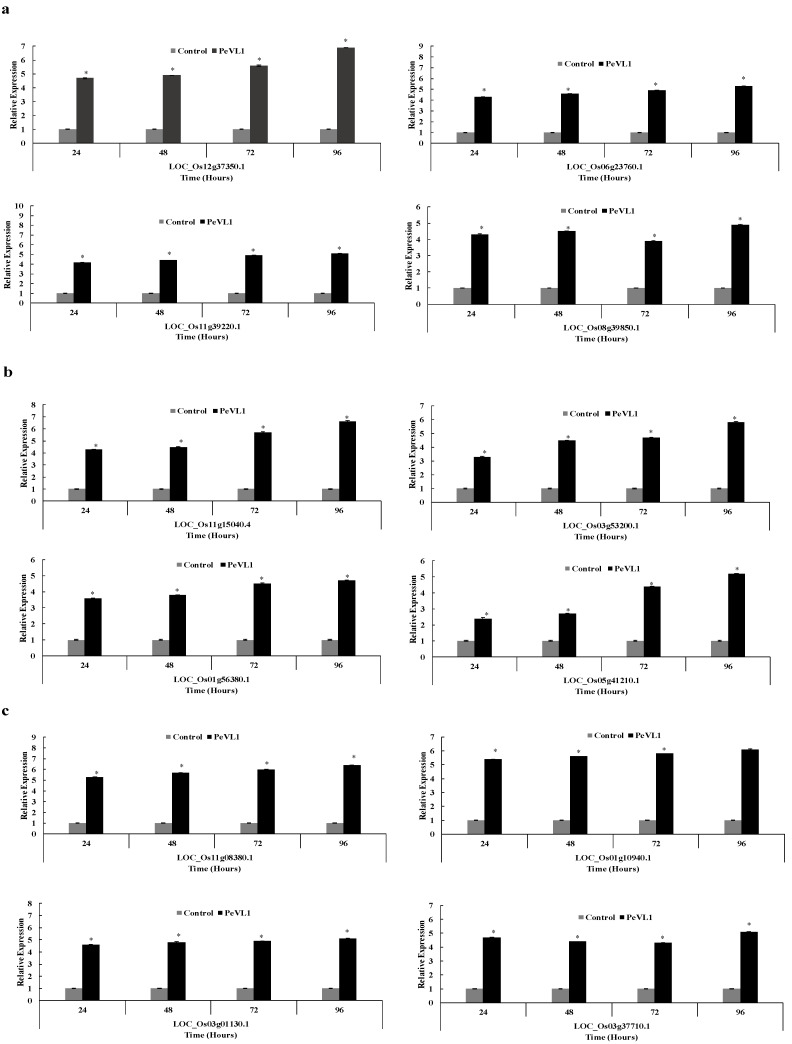
After PeVL1 elicitor treatment and *M. ruralis* infestation, the relative expression of plant defense from the JA, SA and ET pathway, i.e., (**a**–**c**), respectively, was detected. An asterisk next to each gene indicates a significant difference from the buffer control, as determined using Student’s *t*-test (*p* < 0.05).

PeVL1 boosted defenses in *O. sativa* seedlings. PeVL1 treatment slightly upregulated all JA, SA, and ET pathway test genes, respectively (Figure 7a–c). The Log2 of all test genes was calculated using fold-change expression values, indicating that transcription triggered *M. ruralis* confrontation.

## 4. Discussion

A new biological tool for pest control, elicitors share a role in signaling systems and plant defense [13,14,15,16,17,35]. PAMPs and MAMPs are abundant in both necrotrophic or biotrophic pathogenic bacteria and fungi [36]. PeVL1, a protein elicitor, has antimicrobial and biocontrol potential against *M. ruralis*. In *O. sativa* crops, chemical elicitors such as methyl-jasmonate and benzo-thiadiazole, as well as proteinase inhibitors, have been shown to significantly reduce herbivore pest activity. According to previous research, methyl salicylate reduces the population of *Aphis glycines* Matsumura (Hemiptera: Aphididae) by up to 40% [36,37]. In this study, PeVL1 inhibited herbivores by altering plant physical characteristics. Trichomes are the first step in building physical resistance to pathogens and herbivores. These affect herbivore shape and trichome density in *Solanum* spp. Seven trichomes with two major defense-related effects were evaluated [38]. Therefore, for example, thick matte hair on a plant’s surface provides energy while limiting feeding and preventing insect access. Tomato *Solanum hirsutum*, for example, is avoided by *M. ruralis*. The plant’s “pubescence” provides resistance to pests by covering the plant’s surface with epidermal cell appendages of unicellular or multicellular hairs. Soybeans with thick trichomes had lower *Leptinotarsa decemlineata* (Chrysomelidae: Coleoptera) settlement than those with thin trichomes [39]. PeVL1 reduced disease severity by initiating a photosynthetic process [40], and increased induced resistance in *S. lycopersicum* seedlings treated with PeVL1. The physical barrier aids by means of a ration of cell wall expansion and is responsible for plant resilience [41]. The lignin concentration of *Chrysanthemum indicum* increased aphid tolerance [42]. In response to biotic and abiotic stress, plants produce trichomes and wax [43]. A number of exogenous phyto-hormones have been shown to influence trichome density and cuticular wax deposition in *Arabidopsis* and tomatoes [44]. *Brassica napus* has waxy leaves that require SA [45]. SA and JA accumulation increased trichome density and cuticular wax deposition in *O. sativa* plants treated with PeVL1. The PeVL1 elicitor had a negative influence on *M. ruralis* fecundity. PeVL1-treated plants had far fewer *M. ruralis* than the control. Exogenous SA and MJ depletes *M. ruralis* mean fecundity to 50% [36,45]. The lowest *M. ruralis* fecundity was observed at lower temperatures (e.g., 23 °C), while the highest fecundity was observed at (27 °C), attributed to a declining proportion of metabolic activities [46]. The maximum larval development time was observed even at a lower temperature (23 °C), indicating that a one-degree temperature increase had an effect on the insect’s life cycle [47].

After being exposed to PeVL1, *O. sativa* became more resistant to *M. ruralis* than before. A systemic defense response is triggered by beneficial bacteria. This response is controlled by a signaling system that links the plant hormones SA, JA, and ethylene, and it is controlled by the bacteria ET [48]. Some data suggests that the SA, JA, and ET pathways work together to change how the plant responds to different pathogens [48]. These plant defense mechanisms play defensive role against major agricultural pests [28,48]. To activate PR genes via interactions with TGA transcription factors, authors found that PeVL1 increased JA, SA, and ET-responsive genes levels [49]. The data show that PeVL1-mediated systemic defensive responses in *O. sativa* are influenced by relative expression levels. Discoveries from the present work also support former research by Chaerle et al. [50]; secondary metabolite accumulation can help plants fight infection by generating mechanical barriers to pathogen growth, which stimulates phenolic metabolism and lignin synthesis. In addition, phenolic compounds such as scopoletin and phenolic acids can strengthen the cell wall, making it more resistant to bacteria and fungi. This crosslinking of phenyl propanoidesters with ferulic acid results in auto-fluorescent lignin-like polymers, such as hydroxycinnamic acids and their derivatives [51]. Systemic defenses are activated when an *M. ruralis* feeds on plants [52]. These findings suggest that our work helps us better understand how PeVL1 works in *O. sativa* for the management of *M. ruralis* [53].

## 5. Conclusions

PeVL1 made *O. sativa* more resistant to *M. ruralis*, which made the second and third generations of *M. ruralis* less fertile and more likely to have *M. ruralis*. Mechanical defenses played a small role in the resistance characteristics. The surface structure of *O. sativa* leaves changed after PeVL1 was added to the plant. SA, JA, and ET, which are thought to be involved in systemic defense responses in *O. sativa* when PeVL1 is used, showed small increases in relative expression levels. PeVL1 also had a big impact on *M. ruralis* lifespan in the lab, but additional investigation is needed to make it more effective in the field. In our research, we found that PeVL1 may possibly be cast-off as a “vaccine” to protect plants of *O. sativa* against the insect pest, *M. ruralis*.

## Figures and Tables

**Table 1:** Primers for plant defense genes JA, SA, and ET.

JA/SA/ET Pathways	Test Genes	Forward Sequence (5′…… 3′)	Reverse Sequence (5′…… 3′)
JA	*LOC_Os12g37350.1*	CTCCATGGTTGGTGGAACGA	TAGGGGTACTGGCCGAAGTT
JA	*LOC_Os11g39220.1*	GCTCACACTTGCGGAATCAC	GGCTTTGTTTGGGGCAACAT
JA	*LOC_Os06g23760.1*	AGCTCAGGTCACCGACTTTG	ATGAAACGGGAATTCGGCCT
JA	*LOC_Os08g39850.1*	GAGATGAGGAGTTCGCGAGG	ACGGCAAGAAGAGGTCATGG
SA	*LOC_Os11g15040.4*	TTCAATGCAGGAGGGACGAC	AGTCATGCATGCGGTTCTCA
SA	*LOC_Os01g56380.1*	GCATCAACGTCGTGCCTTTC	GATCGGAGCAGTAGACGACG
SA	*LOC_Os03g53200.1*	TCTTCGACAAGAACGGCGAT	AGGCCAAGAGAACGAGTCAC
SA	*LOC_Os05g41210.1*	GCGACGGTTGCATCACTACT	GCCTCAGTTGGGTTCTGACC
ET	*LOC_Os11g08380.1*	TAGCAATGGCCGCTTCAAGA	CTTGAAGCTCGGGTAGTCGG
ET	*LOC_Os03g01130.1*	GCGGAGCTGTACCTCAACAT	CTTGGAAGACTCCGCTGGTT
ET	*LOC_Os01g10940.1*	CGGAGACGTTCCTCTTCACC	CTTCTCGTAGTCGACGCTGG
ET	*LOC_Os03g37710.1*	TGAGAGGAGCCATAGGTGGT	GTAGCGGCTCATGTCGAAGT
	*18S*	GTGACGGGTGACGGAGAATT	GACACTAATGCGCCCGGTAT

## Data Availability

The required data set is already available in manuscript file; other data sets generated during the study are available upon request from corresponding author.
